# Observation of Landau levels on nitrogen-doped flat graphite surfaces without external magnetic fields

**DOI:** 10.1038/srep16412

**Published:** 2015-11-09

**Authors:** Takahiro Kondo, Donghui Guo, Taishi Shikano, Tetsuya Suzuki, Masataka Sakurai, Susumu Okada, Junji Nakamura

**Affiliations:** 1 Faculty of Pure and Applied Sciences, University of Tsukuba, 1-1-1 Tennodai, Tsukuba, Ibaraki 305-8573, Japan; 2 Tsukuba Research Center for Interdisciplinary Materials Science (TIMS) & Center for Integrated Research in Fundamental Science and Engineering (CiRfSE), University of Tsukuba, 1-1-1 Tennodai, Tsukuba, Ibaraki 305-8573, Japan

## Abstract

Under perpendicular external magnetic fields, two-dimensional carriers exhibit Landau levels (LLs). However, it has recently been reported that LLs have been observed on graphene and graphite surfaces without external magnetic fields being applied. These anomalous LLs have been ascribed primarily to a strain of graphene sheets, leading to in-plane hopping modulation of electrons. Here, we report the observation of the LLs of massive Dirac fermions on atomically flat areas of a nitrogen-doped graphite surface in the absence of external magnetic fields. The corresponding magnetic fields were estimated to be as much as approximately 100 T. The generation of the LLs at the area with negligible strain can be explained by inequivalent hopping of π electrons that takes place at the perimeter of high-potential domains surrounded by positively charged substituted graphitic-nitrogen atoms.

In the presence of an external magnetic field, charge carriers of conductive material circulate in cyclotron orbits perpendicular to the field, with quantised energies called Landau levels (LLs). In the case of a two-dimensional electron gas, the energy differences between adjacent LLs are equal, and the quantised energy levels are described by the following equation:


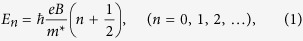


where *n* is the Landau index, *E*_n_ is the energy of the *n*th LL, 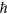 is Plank’s constant, *e* is the electron charge, *B* is the external magnetic field and m* is the effective mass of the carrier arising from interactions with the lattice. The energy differences of adjacent LLs are, however, not the same if the carriers are massless Dirac fermions (DFs)^1^,^2^,^3^,^4^, such as LLs for single-layer graphene, because of the linear dispersion of the electronic structure near the Dirac point (DP)^1^. The LLs of DFs consist of a field-independent state at zero energy followed by a sequence of levels with square-root dependence in both the fields and level index:


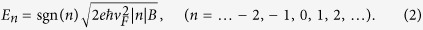


Here, *v*_*F*_ represents the Fermi velocity. When two graphene layers form an AB-stacking bilayer, the carriers are no longer massless but rather massive DFs, i.e., they form a unique electronic structure^5^,^6^. The energy differences of adjacent LLs are thus not the same as those of a two-dimensional electron gas and of graphene. The LLs energies are described as follows^1^,^5^,^6^,^7^:


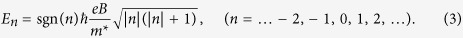


Note that the field-independent zero-energy state is still preserved as in equation (2). In addition to these specific LL characteristics, several peculiar optical and transport properties of bilayer graphene have been reported that are attributable to its unique electronic structure^6^. In the case of a Bernal-stacked graphite surface composed of several graphene layers, the LLs of two-dimensional electron gas, massless DFs, and massive DFs have been reported to appear together or separately under external magnetic fields, based on scanning tunnelling spectroscopy (STS) measurements on highly oriented pyrolytic graphite (HOPG) surfaces^5^,^8^.

The formation of LLs in graphene even without external magnetic fields being applied was theoretically predicted to be possible if a specific structural symmetry break, such as non-uniform shear strain in the lattice, occurred and a pseudo-magnetic field was generated^9^. In such situations, inequivalent hopping between nearest-neighbour (different sublattice) carbon atoms in the tight-binding model has been reported to induce a pseudo-magnetic field. Several groups have subsequently reported using STS to observe LLs without external magnetic fields at the graphene nano-bubble on Pt(111)^10^, the ridges of HOPG^11^, the domain boundary of partially potassium-intercalated graphite^12^, the graphene nano-bubble on Ru(0001)^13^, and the twisted graphene bilayer on Rh foil^14^. In every case, LLs were observed at locations with corrugations. Thus, the observed LLs can be ascribed to strain-induced pseudo-magnetic fields. In the case of partially potassium-intercalated graphite, another possible origin of the pseudo-magnetic field has been proposed because the corrugation at the observed domain boundary is not sufficiently large to explain the observed high pseudo-magnetic field. In the proposed domain model, the pseudo-magnetic fields are ascribed to the gradient of the on-site potential of carbon atoms at the perimeter of the potassium-free domain, which results in inequivalent hopping between the nearest-neighbour carbon atoms. Here, we report STS observations of the Landau levels of massive Dirac fermions on an atomically flat area of a nitrogen-doped graphite surface to which no external magnetic fields were applied. The occurrence of these Landau levels supports the domain model.

## Results

**Figure 1:**
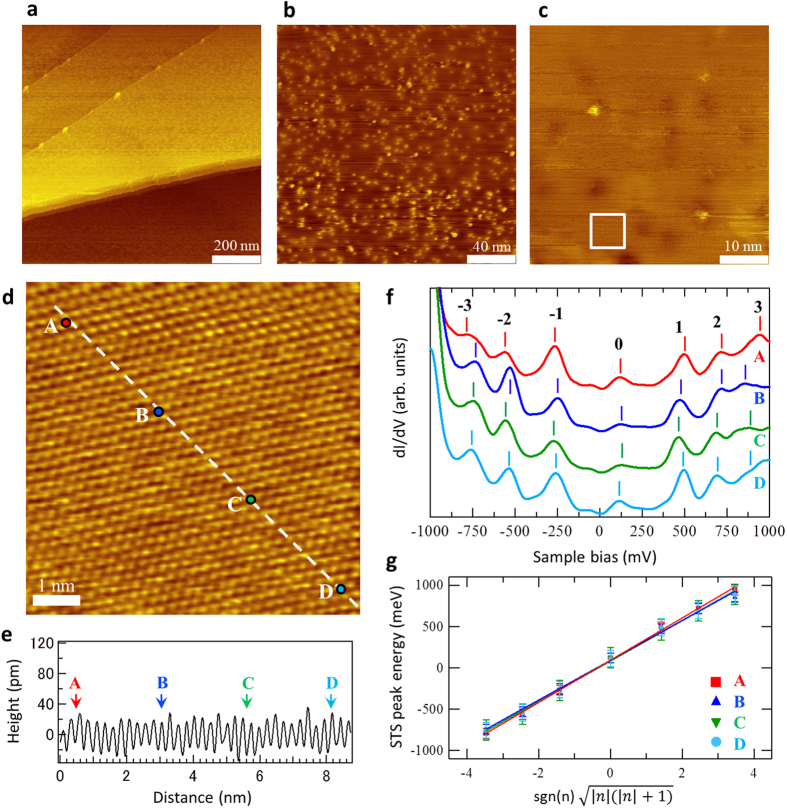
STS spectra at an atomically flat area of the nitrogen-doped graphite surface show Landau levels of bilayer graphene (massive Dirac fermion). (**a–c)** STM images ((**a**) 300 mV, 59.4 pA, 1000 × 1000 nm^2^; (**b**) 300 mV, 59.1 pA, 200 × 200 nm^2^; (**c**) **−**500 mV, 96.6 pA, 50 × 50 nm^2^)). (**d**) STM image at the position shown by the white square in (**c)** (−500 mV, 97.4 pA, 7 × 7 nm^2^). (**e)** Line profile of the white dashed line in (**d**). (**f**) STS obtained at positions labelled A, B, C, and D in (**d**). (**g**) Linear scaling between the peak positions in the STS and 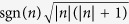. Error bars indicate the variations in ten measurements at each position (see Figure S7).

The nitrogen-doped graphite used in this study was prepared by nitrogen ion bombardment of an HOPG surface at room temperature, followed by annealing at 900 K in an ultra high-vacuum chamber. Scanning tunnelling microscopy (STM) images of this surface are shown in Fig. 1a–d. Nitrogen atoms doped in the graphite surface are imaged as bright or dark spots with a diameter of approximately 3 nm. The differences in the brightness are due to the bonding type of the nitrogen species and can be affected by the sample bias condition, as reported in our previous study^15^. The amount of doped nitrogen was estimated as 0.04 at % by counting the spots in the STM images (Supplementary Figure S1). As the line profile along the atomic arrangement (the dashed line in Fig. 1e) shows, the observed surface region (Fig. 1e) is atomically flat, i.e., there is no apparent corrugation, while there is a sinusoidal curve with the periodicity of *β*-site carbon of Bernal-stacked graphite, 0.245 ± 0.01 nm, which is typically observed at the flat region of the pristine graphite surface by STM^16^. In the STS measurements of the atomically flat region, we observed many distinct peaks, as shown in Fig. 1f, in which STS spectra A–D were measured at the corresponding positions in the STM image shown in Fig. 1d. Similar STS spectra with several distinct peaks were observed reproducibly at various positions on the nitrogen-doped graphite surface at over 300 points (Supplemental Figures S2–S5). However, spectra with other features were also observed in some regions of the surface (over 1500 points). In these latter cases, single peaks near the Fermi level due to the localised states of the non-bonding p_z_ orbitals of carbon atoms^15^, parabolic spectra, or V-shaped spectra were observed. In the case of the nitrogen-doped graphite with a nitrogen concentration higher than 0.04 at %, it was difficult to obtain stable measurements using STM and STS because of the high surface roughness.

The discrete peaks in the STS spectra were found to correspond to the LL peaks of the bilayer graphene. We used equations (1)–(3), 4, 4 to analyse the peaks and found that the best fit was obtained when we used equation (3) (Supplemental Figure S6). The results of the fitting analysis are shown in Fig. 1g, in which the error bars indicate the variation in the peak positions for ten measurements at each position (Supplemental Figure S7). In every case, the fitting accuracy, represented by Pearson’s *r* was greater than 0.998 for the assignment of the STS peak at ~260 meV as the *n* = 0 energy level of the LLs of the bilayer graphene (Supplemental Figure S6). The pseudo-magnetic field *B*_s_ could thus be estimated from the slope of the fitted line as *B*_s_ = 63 ± 3–105 ± 5 T using equation (3) and assuming that the effective mass m* of the bilayer graphene is 0.03–0.05 *m*_e_^17^ (where *m*_e_ is the mass of an electron). The corresponding magnetic length (cyclotron radii) *l* can be derived as 2.5–3.2 ± 0.1 nm by calculating 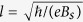. STS spectra with peaks showing LLs were observed at many positions, and more than 90% of the analysed results are in clear agreement with the LLs of the bilayer graphene (Supplemental Figures S4 and S5). On the other hand, LLs of single-layer graphene were also observed at a few positions (Supplemental Figure S8).

**Figure 2:**
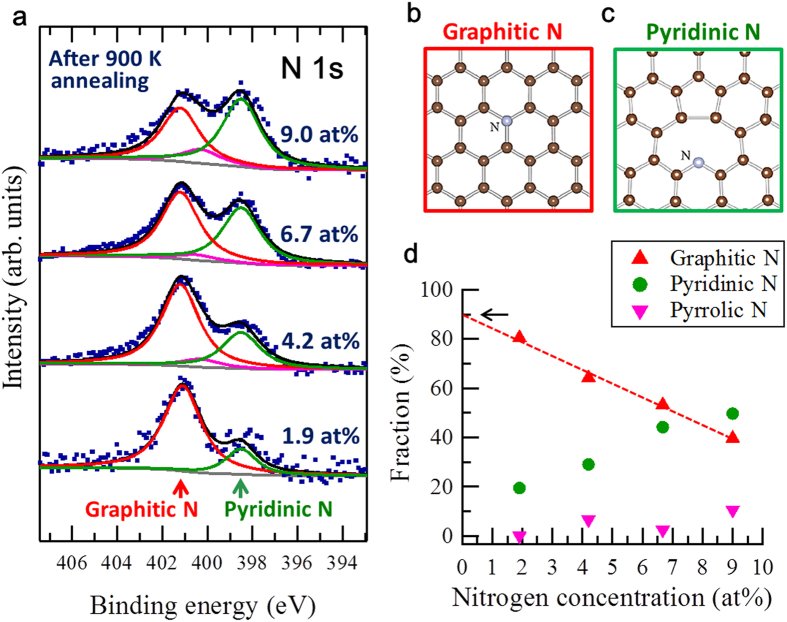
XPS spectra of nitrogen-doped graphite surfaces showing graphitic-N dominance at low N concentration. (**a**) XPS N1s core level spectra of nitrogen-doped graphite surfaces with different N concentrations. (**b,c**) Schematic images of graphitic N and pyridinic N. (**d**) The fractions of graphitic N, pyloric N and pyridinic N in nitrogen as a function of nitrogen concentration of the nitrogen-doped graphite surface.

Although strain-induced LLs under no external magnetic field have been reported in several studies on graphene with corrugation, as described above, the region in which LLs were observed in our current study was indeed atomically flat, and the atomic periodicity was the same as that of pristine graphite, as shown in Fig. 1. The observed LLs on the surface of the nitrogen-doped graphite were thus due not to strains but to electronic modifications resulting from the nitrogen doping. In particular, the potential or charge of carbon atoms near a nitrogen atom may modify π electron systems, depending on the electronic state of the nitrogen. Several types of nitrogen species are known for doped nitrogen in graphitic materials, which modifies the π electron system differently and can be distinguished by their core level signals of nitrogen (N1s) spectra in X-ray photo electron spectroscopy (XPS). The graphitic N species (N bonded to three carbon atoms, also called substituted N or quaternary N) exhibits an N1s binding energy (BE) peak at 401.2 ± 0.2 eV, the pyridinic N species (N bonded to two carbon atoms) exhibits an N1s BE peak at 398.5 ± 0.2 eV, and the pyloric N species (NH in pentagon ring) exhibits an N1s BE peak at 400.3 ± 0.3 eV^18^,^19^,^20^. Figure 2 shows XPS N1s spectra of nitrogen-doped graphite samples with nitrogen concentrations from 1.9 to 9.0 at % (see Supplemental Figure 9). The fraction of graphitic N in nitrogen increases as the concentration of doped nitrogen decreases. Based on the results and by extrapolation, as shown in Fig. 2d, we estimate that the graphitic N fraction is approximately 90% of the sample with N of 0.04 at % used in the STM/STS measurements.

**Figure 3:**
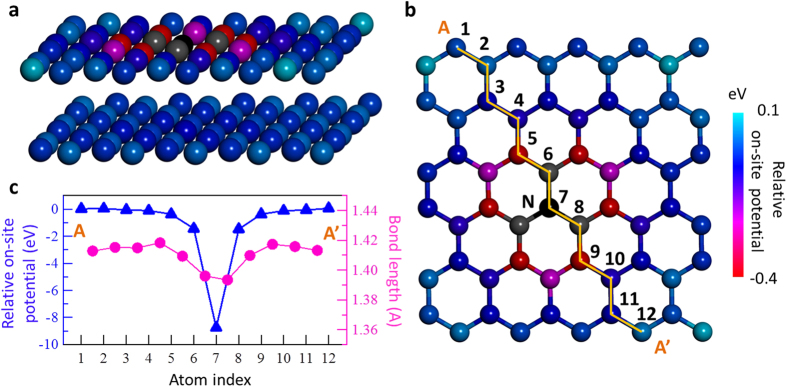
Large on-site potential differences in the nitrogen-doped bilayer graphene surface calculated by DFT. (**a,b**) Structure of bilayer graphene with graphitic-N calculated by DFT. The colour of the atom represents the on-site potential with respect to the carbon of pristine graphene. Graphitic N is the atom labelled 7 in (**b)**. (**c**) Calculated bond lengths and relative on-site potentials at the positions labelled from 1 to 12 in (**b**).

The significant chemical shift in N1s BE for graphitic N (401.2 eV) compared to that for pyridinic N (398.5 eV) indicates positive charge of the graphitic N, given that the N1s BE for N^+^ in pyridinium ion, C_5_H_5_NH^+^, is 401.2 eV^18^,^20^. The positive charge can be explained by electron transfer from graphitic N to the π* states of graphite^15^, which is supported by analysis of the Dirac point^21^,^22^ and theoretical calculations based on density functional theory (DFT)^23^ for nitrogen-doped graphene. Given the positive charge of graphitic N, the neighbouring carbon atoms should be negatively charged, because of the screening effect. The on-site potentials of carbon atoms around graphitic N after screening of the positive charge can then be calculated by DFT for a bilayer graphene with graphitic N species, as shown in Fig. 3. In this study, we put the graphitic N at the α site (the site at which the C atom is located in the lower layer), shown as index 7 in Fig. 3b. The on-site potential of nitrogen is as low as −8.8 eV relative to that for the carbon atoms of the pristine bilayer graphene (0 eV), which mainly corresponds to the positive charge indicated by the high BE of N1s. The on-site potentials of the nearest-neighbour carbon atoms are about −1.5 eV due to insufficient screening of the positive charge of nitrogen. The DFT calculation thus indicates that on-site potential contours are two-dimensionally distributed on a graphene sheet. It is thus expected that π-electron should feel the potential gradient, which influences the hopping of electrons. With respect to the calculated strain due to N implantation, the difference in bond length between C–N and C–C bonds is very small, approximately 0.0025 nm at most, i.e., approximately 1% change. Using the strain model proposed by Guinea *et al.*, the 1% strain has been calculated to produce a pseudo-magnetic field of 7 T^9^, which is much smaller than the estimated pseudo-magnetic field of 60–100 T for the nitrogen-doped graphite.

## Discussion

**Figure 4:**
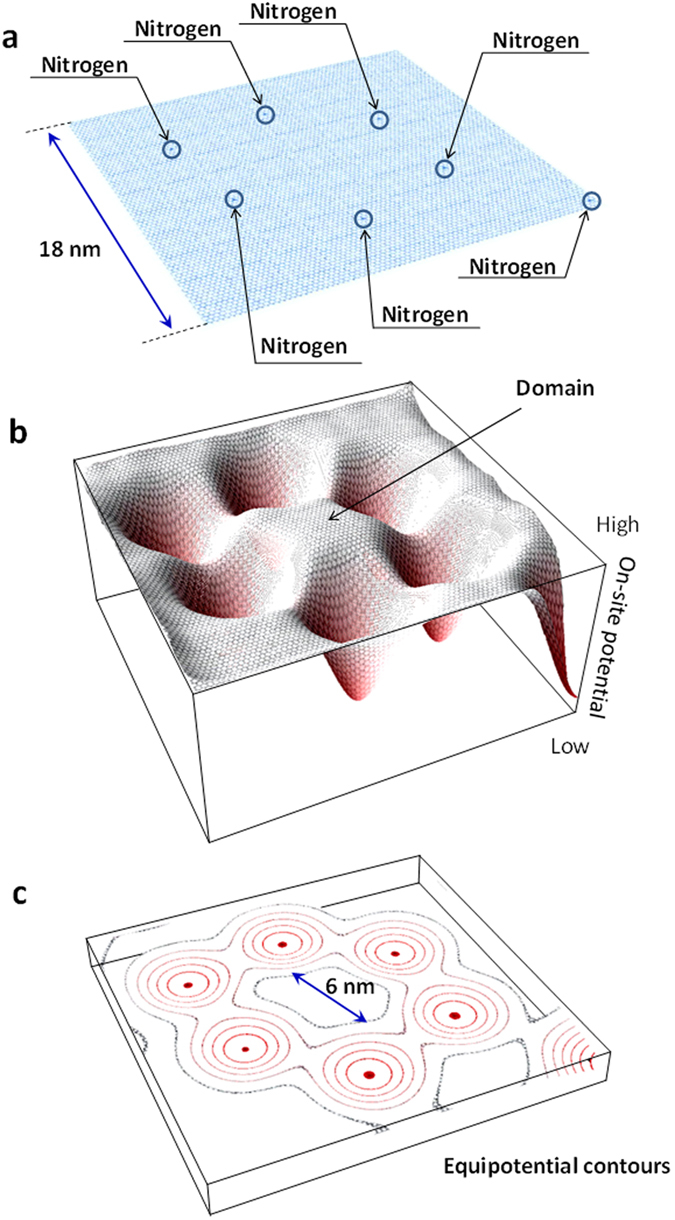
Domain is formed by on-site potential differences around graphitic N. (**a**) Schematic image of the nitrogen-doped graphite surface, where graphitic N is located at the centre positions indicated by arrows. It is atomically flat and there is no large corrugation. (**b**) Schematic image of the on-site potential of carbon at the surface modelled in (**a**). The domain with the higher potential is surrounded by graphitic N, as indicated by the arrow. (**c**) Schematic image of equipotential contours at the surface modelled in (**a**).

Here, we discuss the origin of the LLs observed on the flat nitrogen-doped graphite surface using the domain model proposed previously^12^. As schematically shown in Fig. 4, because of modulation of the on-site potential of carbon atoms by graphitic N, potential domain with high potential centre is considered to be formed. Along the perimeter of the potential domain, equipotential contours should be produced when nitrogen atoms are doped at a suitable concentration of nitrogen in a graphene sheet, leading to equivalent hopping in a contour. As a result, inequivalent hopping between nearest-neighbour carbon atoms leads to the vector potential of an effective gauge field, and the gauge field in turn leads to the generation of a pseudo-magnetic field. The size of the domain corresponds to the magnetic length estimated from the pseudo-magnetic fields (diameter of 5.0–6.4 ± 0.2 nm). It is thus expected that electron hopping becomes dominant across the equipotential contour along the domain perimeter because of the higher potential in the domain. The cyclotron-like motion of the carrier along the domain perimeter may result in LL formation on the flat nitrogen-doped graphite surface without the application of any external magnetic fields. We consider that both clockwise and anti-clockwise cyclotron motions are present and that net real magnetic field should be zero. It has been reported that additional DPs appear by the periodic potential of a super lattice in graphene, where the energy positions of the additional DPs are determined by the periodicity of the super lattice^24^,^25^. On the other hand, the DPs shift for LLs in the present work is determined by the on-site potential as reported in our previous report^12^. It is thus considered that the origin of the LLs is different from that of the new DPs.

As for the reason why primarily bilayer graphene LLs appear rather than graphene LLs or graphite LLs, the decoupling of the bilayer graphene from the bulk HOPG should be considered. Because our sample was prepared by nitrogen ion bombardment, decoupling of a few layers of the HOPG surface may occur as a result of the bombardment. Subsequent annealing to 900 K most likely causes re-stacking of the decoupled layers to form the nitrogen-doped bilayer graphene at the HOPG surface. The other possible cause of the decoupling is coupling between the first and second graphene layers of the HOPG due to the presence of nitrogen. For example, if graphitic N is located at the α site, the interactions of the p_z_ orbitals of C and N change the layer–layer interaction, compared to that of pristine graphite. Enhancement of the coupling between the first and second graphene layers can also be realized if the charge transfer occurs not only from nitrogen atom to the π* states but also to the interlayer band^26^ of C3s.

## Methods

### Preparation of nitrogen-doped graphite

The fresh HOPG (ZYA-grade, Panasonic Inc.) samples were cleaved in air using adhesive tape, put into an ultra-high-vacuum (UHV) chamber, and annealed at 900 ± 50 K. The HOPG samples were then bombarded by N_2_^+^ ions of 200 eV at the normal incidence using a commercial ion-gun (ANELVA, 5-KV ion gun) at room temperature. The total ion dose applied to the sample was set to be 1.9 × 10^12^ ions/cm^2^ for the samples used for STM and STS, as measured independently using a Faraday cup. After the bombardment, the samples were annealed at 900 K for 300 sec (some of which were annealed for 600 sec). The sample temperature during heating was measured using an infrared thermometer.

### STM and STS measurements

STM/STS measurements were performed in a UHV chamber (UNISOKU, USM-1200) with a base pressure of 1 × 10^−10^ Torr. An SPM 1000 from RHK Technologies was used as the STM controller. The nitrogen doping of graphite samples were conducted in the preparation chamber of the STM apparatus. The samples were cooled down to 5 K using liquid He. STM images were recorded in constant-current mode with a PtIr tip (Pt:Ir = 8:2). STS measurements were carried out by measuring the differential conductance (*dI/dV*) with a lock-in technique using a 1.0-kHz alternating-current (AC) modulation voltage with an amplitude of 20–100 mV. Each STS data point in the figure represents an averaged spectrum of ten spectra measured at the same position. Measurements were conducted for three different nitrogen-doped graphite surfaces to check the reproducibility of the experimental results.

### XPS measurements

The XPS measurements were carried out using JEOL, JPS 9010 TR (X-ray source Al Kα 1486.6 eV, pass energy 50 eV or 10 eV, energy resolution 0.635 eV, estimated from Ag 3d_5/2_ peak width of a clean Ag sample, uncertainty of binding energy ±0.05 eV). The XPS peak energy was calibrated using the peak position of C1s (284.6 eV) of the clean HOPG surface. The nitrogen-doped graphite samples used for the XPS measurements were prepared in the preparation chamber of the XPS apparatus. The nitrogen concentration (at %) was calculated as follows: {(peak area of N1s)/(sensitivity factor of N1s for Al Kα:7.5129)}/{(peak area of C1s)/(sensitivity factor of C1s for Al Kα:4.2586) + (peak area of N1s)/(sensitivity factor of N1s for Al Kα:7.5129)} × 100, where the Shirley background was subtracted from each peak prior to estimation of the area of the peak, using the XPSPeak4.1 software. The fitting analysis was conducted using the Gaussian–Lorentz function (Gaussian:Lorentz ratio = 2:8) using the XPSPeak4.1 software. We set the full width at half maximum of each peak as 2.0 eV to fit the results well.

### DFT calculations

All calculations were performed within the framework of the density functional theory (DFT) using the Simulation Tool for Atom Technology (STATE). We used a local density approximation to treat the exchange-correlation interaction among the interacting electrons, using the Perdew–Zunger functional form to fit the Ceperley–Alder numerical results. Ultrasoft pseudopotentials were adopted to describe the electron–ion interaction generated by using the Vanderbilt scheme. The valence wave function and charge density were expanded in terms of the plane wave basis set with cutoff energies of 25 and 225 Ry, respectively. We considered the bilayer graphene (with each graphene layer having 5 × 3√3 lateral periodicity) and one of the carbon atoms at the α site to be substituted by a nitrogen atom to simulate nitrogen-doped graphene. For this unit cell, the N concentration in the doped graphene was 1.6 at %. Integration over the two-dimensional Brillouin zone (BZ) is carried out using equidistant k-points sampling with 2 × 2 k point in the rectangular BZ. The atomic structures were fully optimised under a zero electric field until the force acting on each atom was less than 5 mRy/Å.

## Additional Information

**How to cite this article**: Kondo, T. *et al.* Observation of Landau levels on nitrogen-doped flat graphite surfaces without external magnetic fields. *Sci. Rep.* **5**, 16412; doi: 10.1038/srep16412 (2015).

## Supplementary Material

Supplementary Information
