# Supplementary material for: Observation of Landau levels on nitrogen-doped flat graphite surfaces without external magnetic fields
Source: Sci Rep. 2015 Nov 9;5:16412. doi: 10.1038/srep16412 (PMC4637907; doi:10.1038/srep16412)
Supplement: Supplementary Information [file srep16412-s1.pdf]

Supplementary Information

Observation of Landau levels on nitrogen-doped flat graphite surfaces without external magnetic fields

Takahiro Kondo[1,2], Donghui Guo[1], Taishi Shikano[1], Tetsuya Suzuki[1], Masataka Sakurai[1], Susumu Okada[1], and Junji Nakamura[1,2]*

* Corresponding author: nakamura@ims.tsukuba.ac.jp

[1]Faculty of Pure and Applied Sciences, University of Tsukuba, 1-1-1 Tennodai, Tsukuba, Ibaraki 305-8573, Japan.

[2]Tsukuba Research Center for Interdisciplinary Materials Science (TIMS) & Center for Integrated Research in Fundamental Science and Engineering (CiRfSE), University of Tsukuba, 1-1-1 Tennodai, Tsukuba, Ibaraki 305-8573, Japan.

S1. How to estimate the nitrogen concentration on the surface

S2. Examples of STS with many peaks

S3. Peak analysis method

S4. LLs of graphene observed on the nitrogen-doped graphite surface

S5. XPS N1s and C1s core level spectra

S1. How to estimate the nitrogen concentration on the surface

The nitrogen concentration was estimated by counting the dark and bright spots visible in each STM image. The eighteen STM images used for the estimation are shown in Figure S1. In each case, the original STM image is shown on the left, while the STM image with pink circles, used to count the spots in STM image is shown on the right. The nitrogen concentration was estimated assuming that a single nitrogen atom is represented by one pink circle with a diameter of 3 nm. The density of the circles in the STM image was divided by the density of carbon atoms per unit area (3.82×10^{15} (atoms/cm^2)) to determine the concentration.

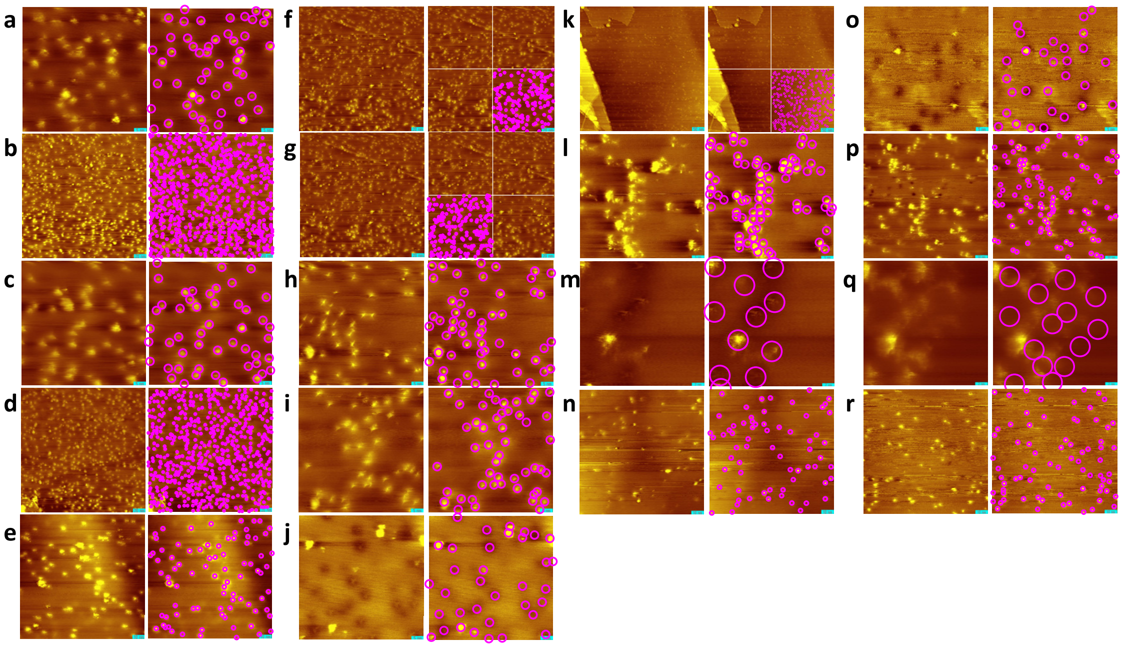

Figure S1 | STM images used for the estimation of nitrogen concentration.
STM images of the nitrogen-doped graphite surface. In each figure, the image on the left is the raw STM image, and image on the right is the STM image with pink circles used to count the spots in the image (see text) **a,** 300 mV, 60 pA, 50 × 50 nm^2, **b,** 300 mV, 60 pA, 200 × 200 nm^2, **c,** 300 mV, 60 pA, 50 × 50 nm^2, **d,** 300 mV, 60 pA, 200 × 200 nm^2, **e,** 500 mV, 180 pA, 100 × 100 nm^2, **f,** 300 mV, 180 pA, 200 × 200 nm^2, **g,** 300 mV, 180 pA, 200 × 200 nm^2, **h,** 300 mV, 60 pA, 50 × 50 nm^2, **i,** 300 mV, 60 pA, 50 × 50 nm^2, **j,** -500 mV, 40 pA, 50 × 50 nm^2, **k,** 200 mV, 180 pA, 500 × 500 nm^2, **l,** 500 mV, 98 pA, 50 × 50 nm^2, **m,** 500 mV, 98 pA, 20 × 20 nm^2, **n,** 500 mV, 68.7 pA, 100 × 100 nm^2, **o,** -500 mV, 96.6 pA, 50 × 50 nm^2, **p,** 500 mV, 98 pA, 100 × 100 nm^2, **q,** 500 mV, 98 pA, 20 × 20 nm^2, and **r,** 500 mV, 97.2 pA, 100 × 100 nm^2.

S2. Examples of STS with many peaks

In addition to those shown in Figure 1 in the main text, we obtained STS spectra with many distinct peaks reproducibly at different positions on the nitrogen-doped graphite surface at over 300 points. Examples are shown in Figures S2–S5. In Figure S2, the STS spectra labelled 1–64 were taken at the positions labelled 1–64 in the STM image. Many peaks appear in the STS spectra, except for spectra labelled 4 and 17. In Figure S3, the STS spectra labelled A–P were taken at the positions labelled A–P in the STM image. All of the STS spectra exhibit many distinct peaks, independent of the measurement position. Figures S4 and S5 show STS results together with atomic resolution STM images. The surface was found to be atomically flat in each case. The STS spectra labelled A–D were taken at the positions labelled A–D in the STM image. In every case, the STS peaks in Figures S4 and S5 were found to correspond to the LLs of bilayer graphene, based on the fitting analysis (Figures S4d and S5g-S5j), as in the case of Figure 1 in the main text.

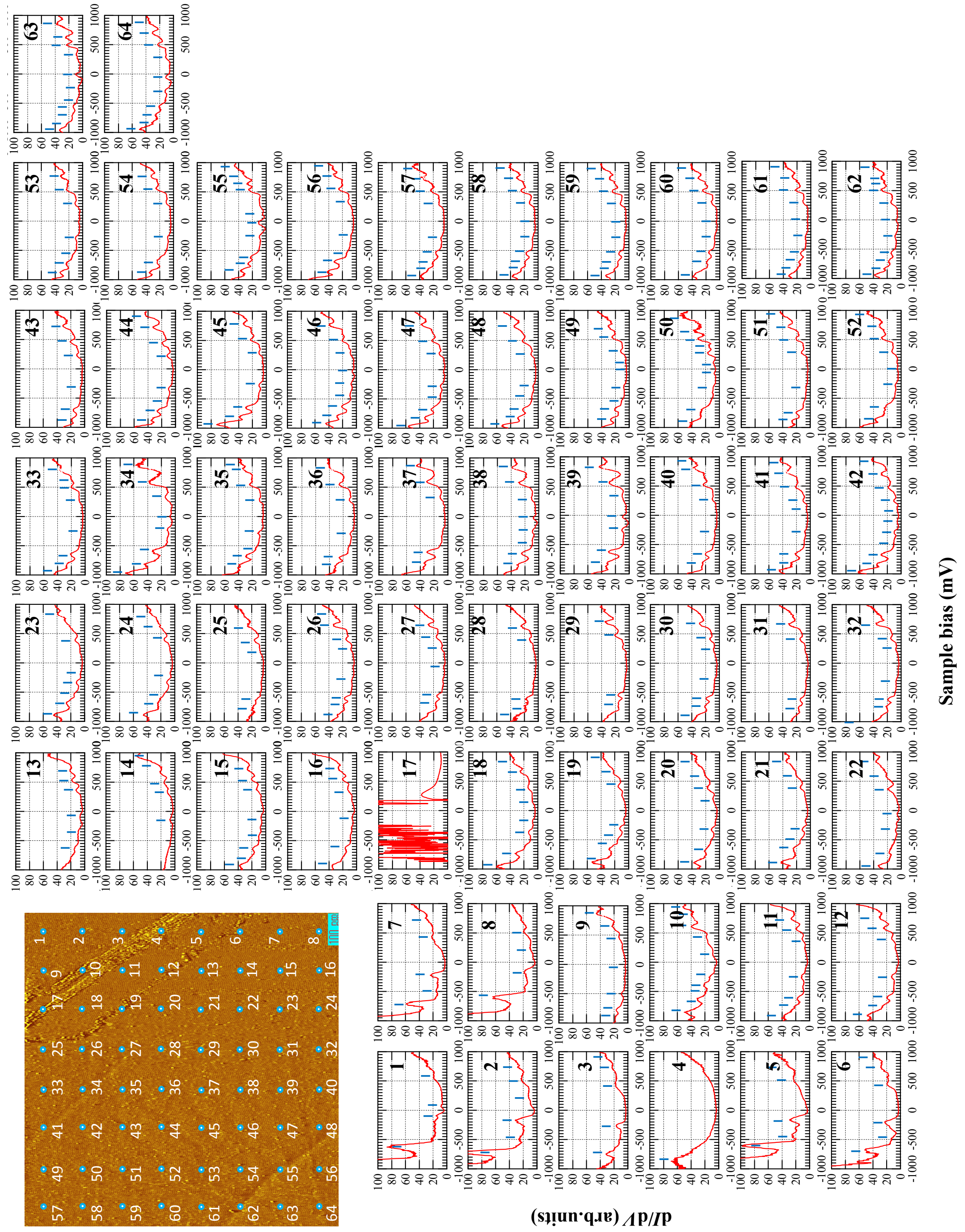

Figure S2 | STS spectra on the nitrogen-doped graphite show many peaks.

STM current image at 5 K (1000 mV, 180 pA, 1000 × 1000 nm^2) and STS spectra. STS spectra of 1–64 were taken at the corresponding positions in the STM image.

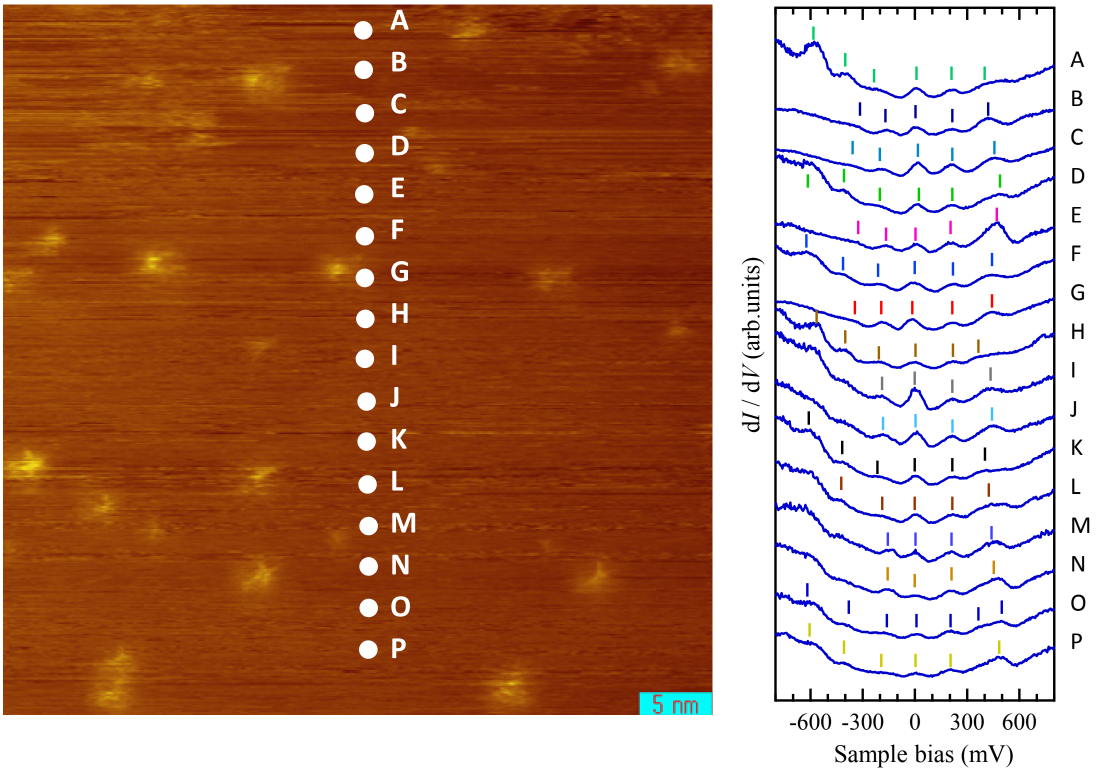

Figure S3 | STS spectra on the nitrogen-doped graphite show many peaks.
STM current image at 5 K (800 mV, 100 pA, 50 × 50 nm^2) and STS spectra. STS spectra at A–P were taken at the corresponding positions in the STM image.

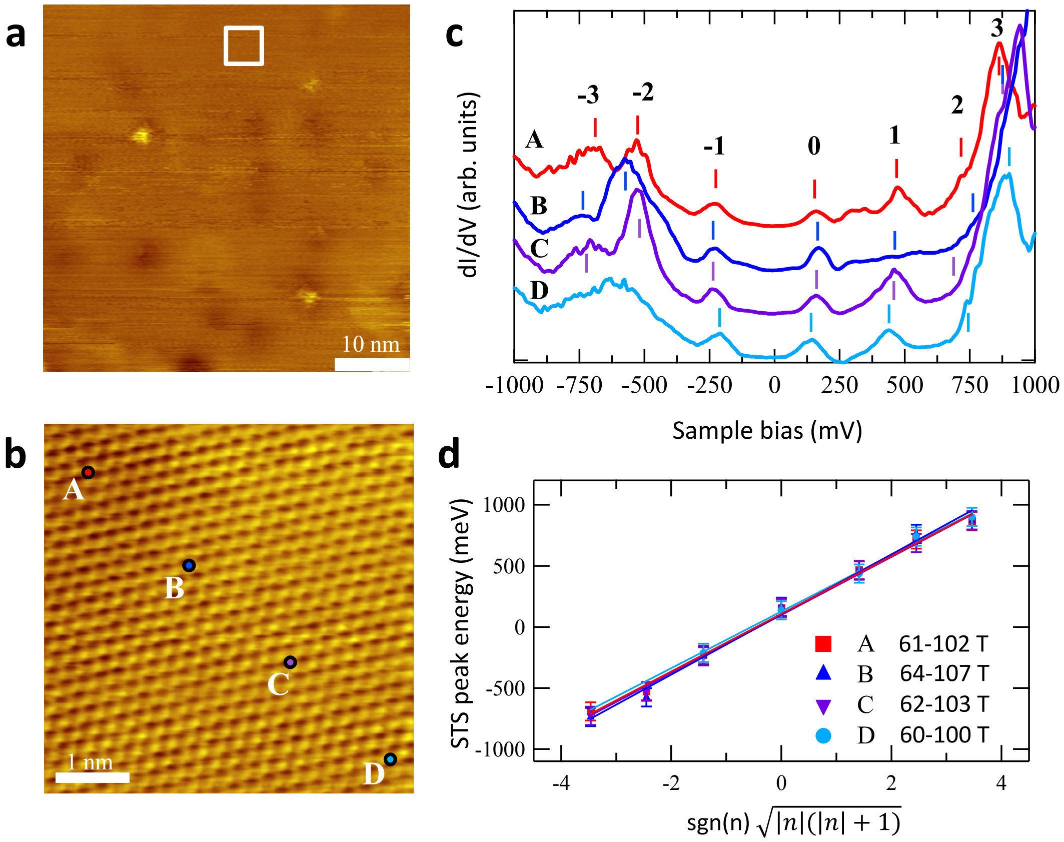

Figure S4 | STS spectra at the atomically flat area of the nitrogen-doped graphite shows Landau levels of bilayer graphene (Different example from Figure 1).

a, STM image (same image as in Figure 1c, -500 mV, 96.6 pA, 50 × 50 nm^2). **b,** STM image at the position indicated by the white square in **a** (-500 mV, 97.4 pA, 5 × 5 nm^2), **c,** STS obtained at the positions labelled A, B, C, and D in **b**. **d,** Linear scaling between the peak positions in STS and $\mathrm{sgn}(n)\sqrt{|n|(|n|+1)}$. The estimated pseudo-magnetic fields are also shown in the figure. We assumed that the effective mass m^* of the bilayer graphene was 0.03–0.05 m_e[17]. Error bars indicate the variation in ten measurements at each position.

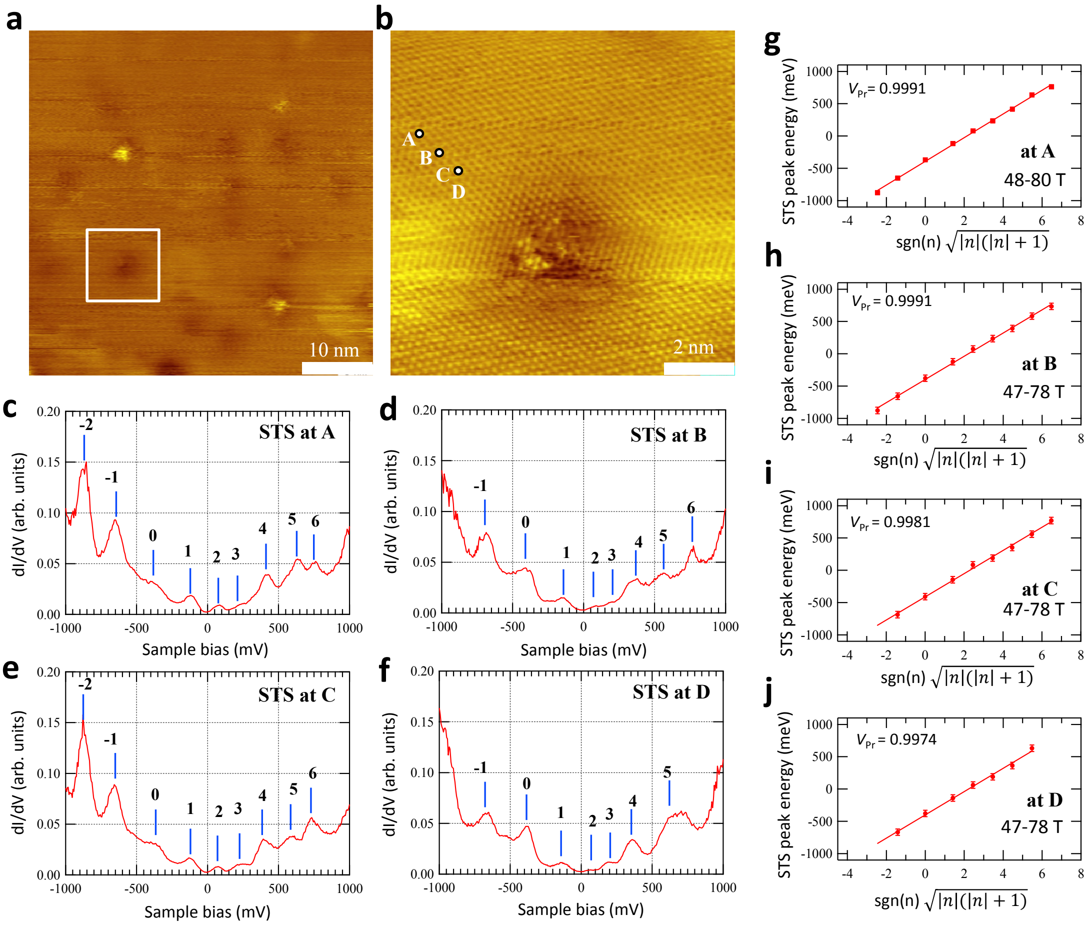

Figure S5 | STS spectra at the atomically flat area of the nitrogen-doped graphite show Landau levels of bilayer graphene (Different example from Figure 1).

a, STM image (same image as in Figure 1c, -500 mV, 96.6 pA, 50 × 50 nm^2). **b,** STM image at the position indicated by the white square in **a** (-500 mV, 94.3 pA, 10 × 10 nm^2). **c-f,** STS obtained at the position labelled A, B, C, and D in **b**. **g-j,** Linear scaling between the peak positions in the STS and $\mathrm{sgn}(n)\sqrt{|n|(|n|+1)}$. The estimated pseudo-magnetic fields are also shown in the figure. We assumed that the effective mass m^* of the bilayer graphene was 0.03–0.05 m_e[17]. Error bars indicate the variation in ten measurements at each position.

S3. Peak analysis method

We conducted a fitting analysis of the STS peak energies using equations (1)-(3) following the procedure described in our previous report[12]. Briefly, values of Pearson's *r* obtained for every possible peak assignment was compared after fitting the results with equations (1)-(3). The best fitting results, i.e. the most probable peak assignments (indicated by the largest Pearson's *r* value) were identified. Figure S6 shows a comparison of Pearson's *r* for each fitting case, e.g., Pearson's *r* for the fitting results for A in Figure 1f are plotted in A of Figure S6. In the case of A in Figure S6, the largest Pearson's *r* was 0.9991, indicated by the solid green circles at -3 on the horizontal axis. The Landau index from the left of the STS spectrum (-790.9 mV) A in Figure 1f was then assigned a value of n = -3 in equation (3). The error bar for each STS peak energy position was determined as the amount of variation in ten measurements. For example, in the case of STS spectrum A in Figure 1f, the peak at -270 mV has a range of variation of 60 mV, as shown in Figure S7, and thus the size of the error bar was set as 60 mV.

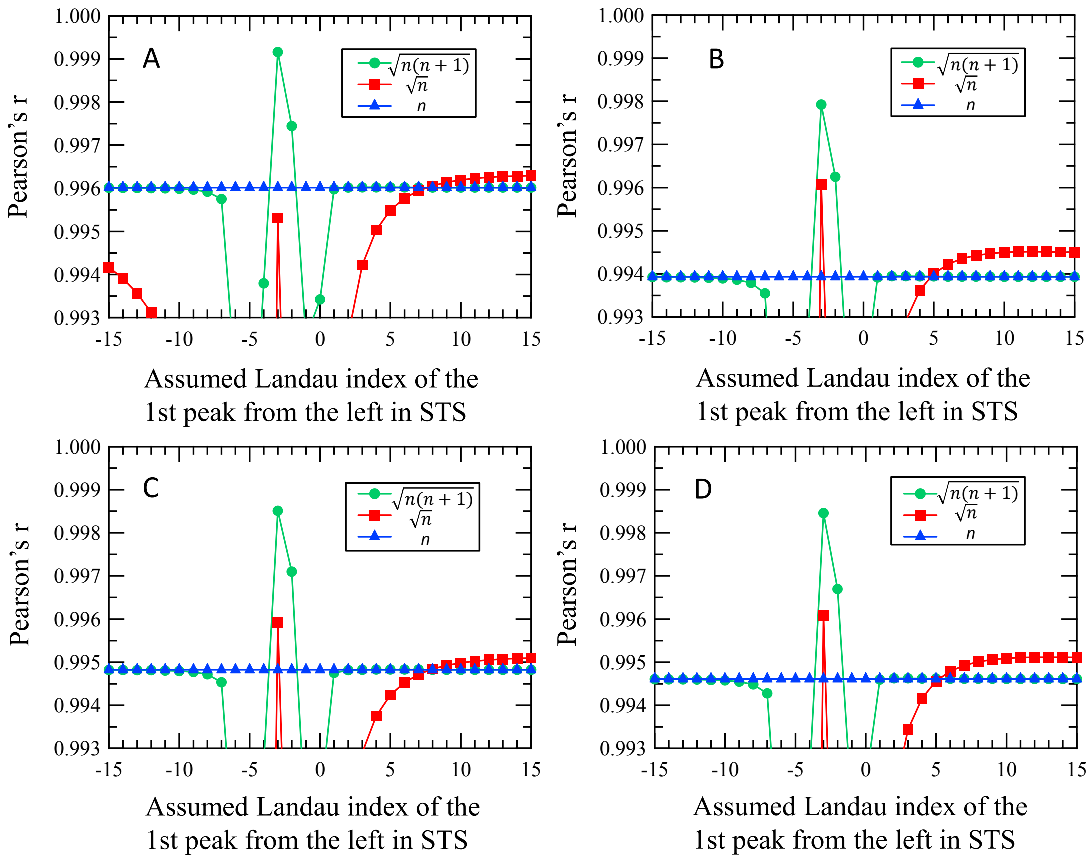

Figure S6 | Comparison of Pearson's r in fitting analysis of STS peak energies.
Pearson's r is plotted as a function of assumed Landau index of the 1st STS peak from the left in each of the spectra A–D in Figure 1f.

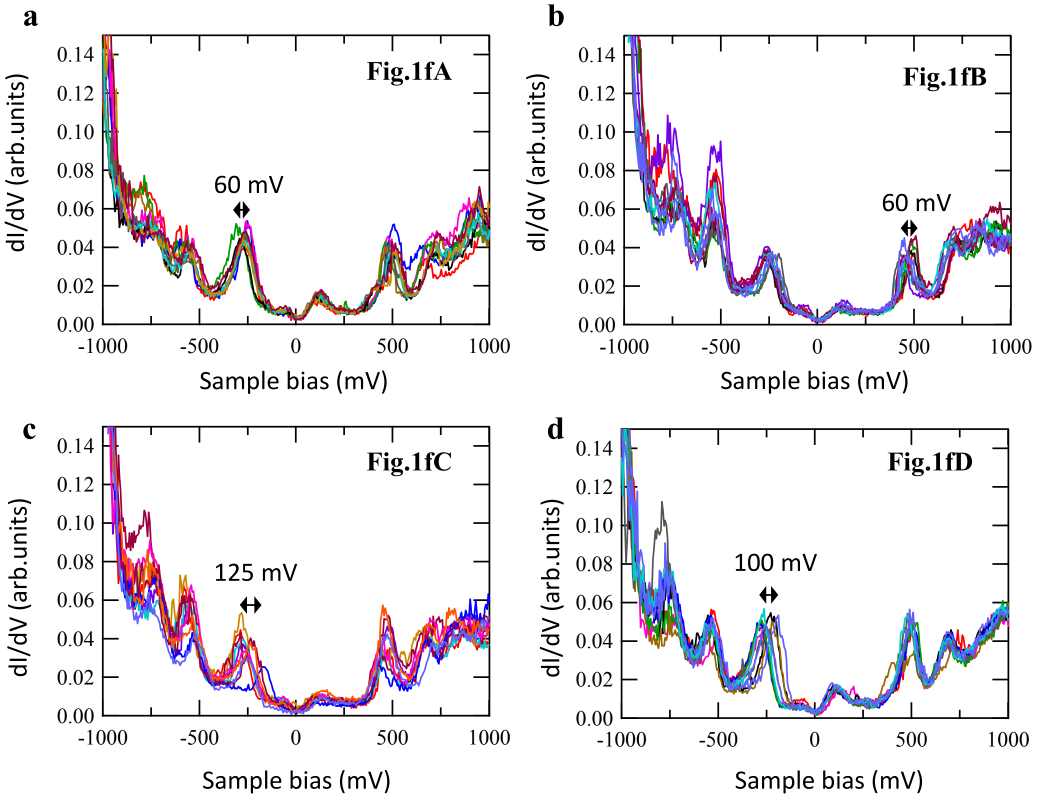

Figure S7 | Raw data sets of STS spectra in Figure 1f.
Ten STS spectra obtained at the positions labelled A, B, C, and D in Figure 1d are shown. The averaged spectrum for ten spectra is shown for each case in Figure 1f. There is a finite variation of approximately 60–125 mV in the peak. The variation is represented by an error bar around the peak position in Figure 1g.

S4. LLs of graphene observed on the nitrogen-doped graphite surface

As described in the main text, STS peaks obtained at the nitrogen-doped graphite surface were found to be correspond primarily to the LLs of the bilayer graphene. However, a few STS spectra were found to correspond to the LLs of graphene. Two examples are shown in Figure S8. The best fitting results for the STS peak energies were obtained for the case in which we used equation (2) (LLs for graphene), and the first peak from the left in the STS spectrum was assumed to correspond to n = -5 and n = -4 for Figs. S8b and S8f, respectively, as indicated by the values of Pearson's r in Figs. S8d and S8h.

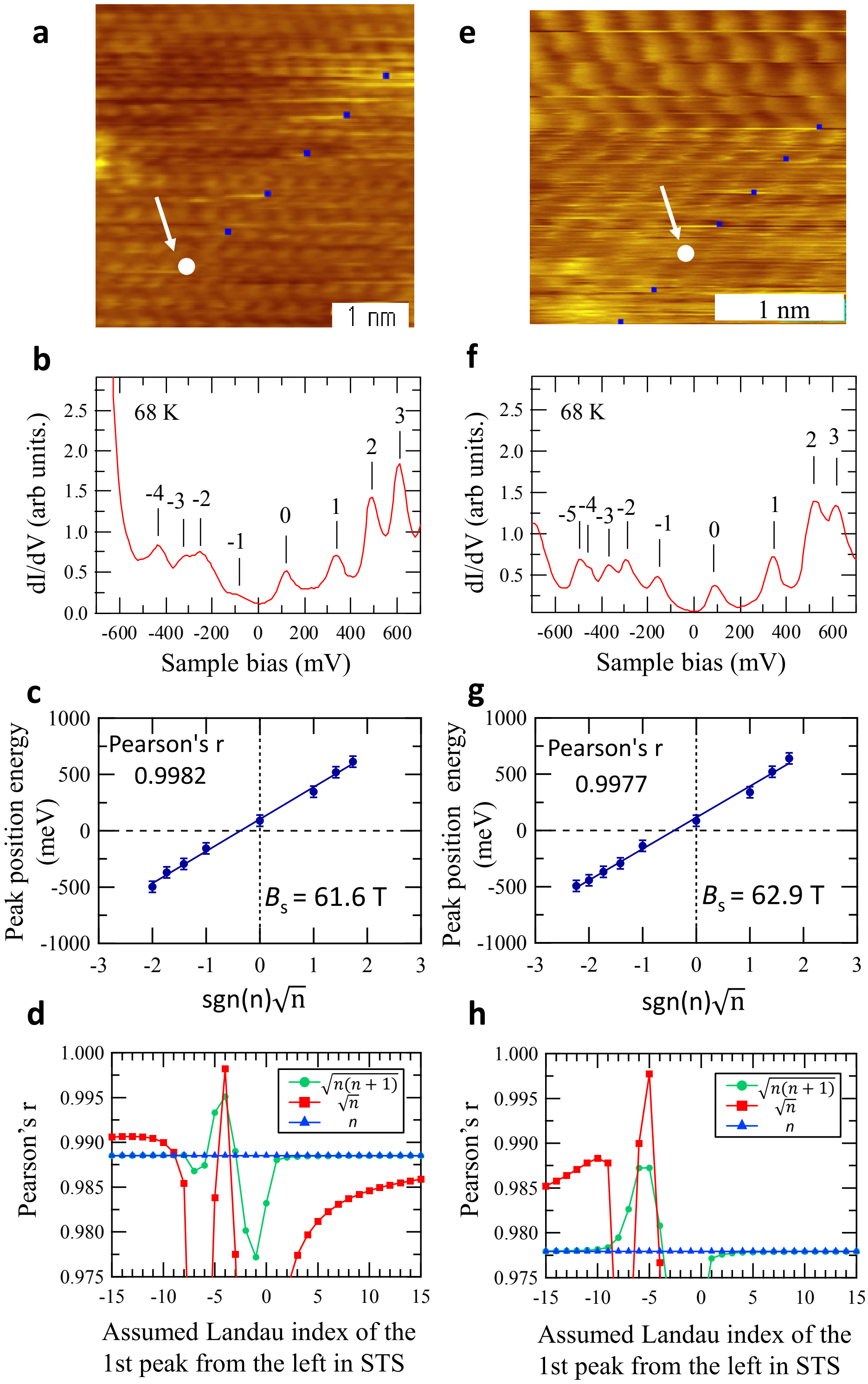

Figure S8 | STS spectra of the nitrogen-doped graphite showing LLs of graphene.
a, e, STM image taken at 68 K (-200 mV, 100 pA). **b, f,** STS obtained at the position indicated by arrow in **a**. **c, g,** Linear scaling between the peak positions in STS and $\sqrt{n}$. **d, h,** Pearson's *r* for the fitting analysis of STS peaks shown in **c** and **g** (see S3).

S5. XPS N1s and C1s core level spectra

Figure S9 shows XPS C1s core level spectra used for the estimation of nitrogen concentrations. Corresponding XPS N1s core level spectra (which are the same spectra shown in Fig. 2) are also shown. C1s peaked at 284.60 eV, 284.65 eV, 284.80 eV, and 284.65 eV for the samples with nitrogen concentration of 1.9 at %, 4.2 at %, 6.7 at %, and 9.0 at%, respectively. Except for the sample with the nitrogen concentration of 1.9 at %, the binding energy of C1s shifted slightly to higher energy, compared to the C1s of the pristine graphite (284.60 eV). This is most likely due to the charge transfer from nitrogen to carbon, which raises the Fermi level.

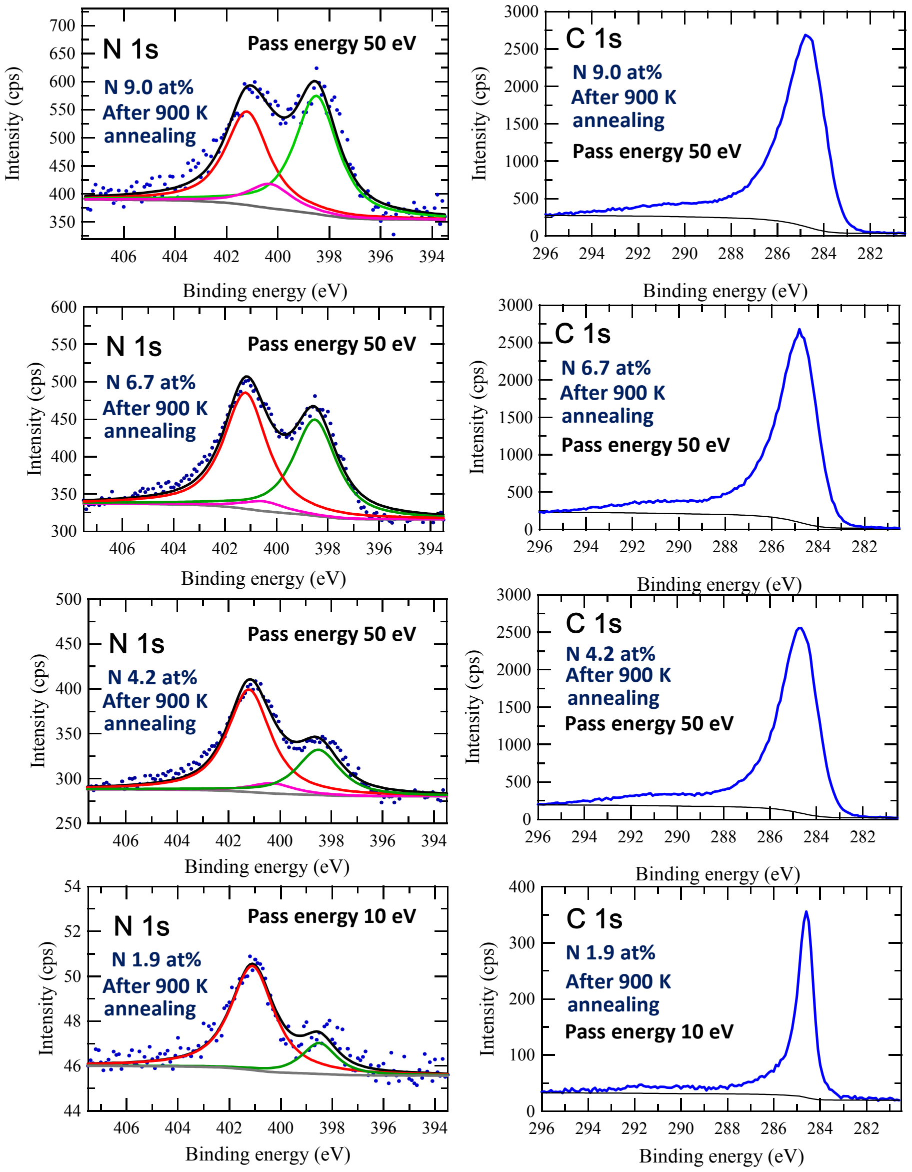

Figure S9 | XPS N1s and C1s core level spectra.

XPS N1s and C1s core level spectra of nitrogen-doped graphite with different nitrogen concentration (9.0, 6.7, 4.2, and 1.9 at %) are shown. All N1s spectra shown here are the same with Figure 2a. Only for the 1.9 at %, the pass energy is selected as 10 eV to clearly resolve the different nitrogen species.
